# Detection of Embryonic Suspensor Cell Death by Whole-Mount TUNEL Assay in Tobacco

**DOI:** 10.3390/plants9091196

**Published:** 2020-09-12

**Authors:** Ce Shi, Pan Luo, Peng Zhao, Meng-Xiang Sun

**Affiliations:** State Key Laboratory of Hybrid Rice, College of Life Sciences, Wuhan University, Wuhan 430072, China; shice@whu.edu.cn (C.S.); panluo@whu.edu.cn (P.L.); mxsun@whu.edu.cn (M.-X.S.)

**Keywords:** tobacco, embryogenesis, suspensor, programmed cell death, TUNEL

## Abstract

Embryonic suspensor in angiosperms is a short-lived structure that connects the embryo to surrounding maternal tissues, which is necessary for early embryogenesis. Timely degeneration via programed cell death is the most distinct feature of the suspensor during embryogenesis. Therefore, the molecular mechanism regulating suspensor cell death is worth in-depth study for embryonic development. However, this process can hardly be detected using conventional methods since early embryos are deeply embedded in the seed coats and inaccessible through traditional tissue section. Hence, it is necessary to develop a reliable protocol for terminal deoxynucleotidyl transferase (TdT) dUTP Nick-End Labeling (TUNEL) analysis using limited living early embryos. Here, we provide a detailed protocol for the whole-mount detection of suspensor cell death using a TUNEL system in tobacco. This method is especially useful for the direct and rapid detection of the spatial-temporal characters of programed cell death during embryogenesis, as well as for the diminishment of the artifacts during material treatment by traditional methods.

## 1. Introduction

The suspensor is a terminally differentiated embryonic structure, which connects the embryo to surrounding endosperms and seed coats in plants and is necessary for embryonic development by transporting nutrients and hormones from the mother tissues to the embryo [1,2,3]. A well-known characteristic of the suspensor is the timely initiation of programmed cell death (PCD) [4,5]. During this process, some classic markers of eukaryotic PCD have been observed in suspensor PCD, such as DNA fragmentation, nuclear degradation, and caspase-like activities [4,6,7,8,9,10,11,12]. Therefore, suspensor has been considered as an ideal model to investigate the molecular mechanism of PCD in plant development [2]. However, because embryos are deeply embedded in the maternal tissues, it is difficult to observe the spatial and temporal dynamics of suspensor PCD directly by conventional methods. Although the methods for the detection of suspensor PCD have been established for years in a few plants with a huge suspensor structure, such as *Picea abies* [6,7,8,9], *Vicia faba* [10], and *Phaseolus coccineus* [11,12]. As the dynamic in situ signals of PCD at the single cell level become more and more important, the establishment of a suitable technique to meet these requirements is obviously needed.

It was previously described that terminal deoxynucleotidyl transferase (TdT) dUTP Nick-End Labeling (TUNEL) is an assay to detect broken DNA fragmentation in situ [13]. This method depends on the template-independent identification of blunt ends of double stranded DNA breaks by TdT. Then, the enzyme catalyzes the addition of fluorescein labeled nucleotides to the 3′-hydroxyl termini of DNA ends, which can be visualized by fluorescence microscopy [14]. For example, to investigate integument tapetum PCD in tobacco, this tissue-specific PCD has been detected by sectioning-based TUNEL assay [15]. During the past decade, we have discussed a series of works about suspensor PCD in tobacco [3,16,17]. Combined with our isolation technique of living early embryos [18], here, we describe a detailed protocol for the whole-mount detection of suspensor PCD using a TUNEL system. Due to its visualization and convenience, this method will be not only widely applied in the determination of the spatial-temporal characters of suspensor PCD during whole process of embryonic development in plants; it also will be helpful for detecting the embryonic cell viability in mutants with abortive embryos.

## 2. Results

### 2.1. Preparation of Hand-Made Tools

**Figure 1:**
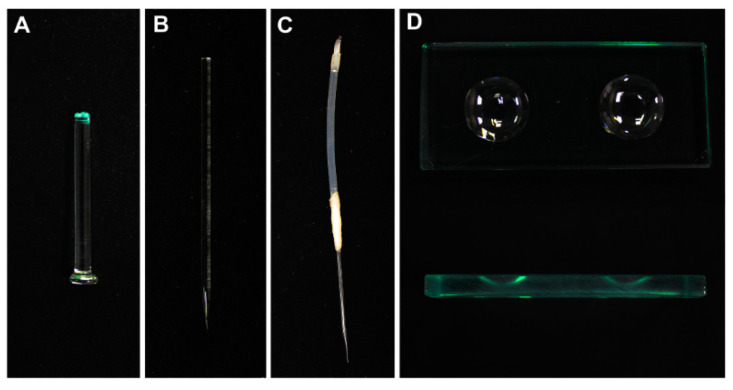
Tools used for the isolation of living tobacco embryos and the terminal deoxynucleotidyl transferase (TdT) dUTP Nick-End Labeling (TUNEL) assay. (**A**) A hand-made glass pestle; (**B**) a hand-made glass needle; (**C**) a hand-made capillary pipette sealed with the latex tubing; (**D**) a thick glass slide with double well concavity.

Isolation of embryos is helpful for direct observing the initiation of suspensor PCD. To date, isolation of tiny early embryos still remains technically challenging. Only a few studies reported the methods for the isolation of early embryos using either laser-capture microdissection (LCM) [19] or manual isolation [19,20]. However, the LCM equipment is not commonly available, and not suitable for isolating living early embryos. To establish a convenient and reliable protocol for isolating early embryos, we developed a set of hand-made tools for the micromanipulation; see Figure 1A–C. Among them, hand-made glass needles (Figure 1B) and the hand-made capillary pipette with latex tubing (Figure 1C) are critical for embryo isolation. Here, we describe the manual preparation of these key tools in detail. 

**Figure 2:**
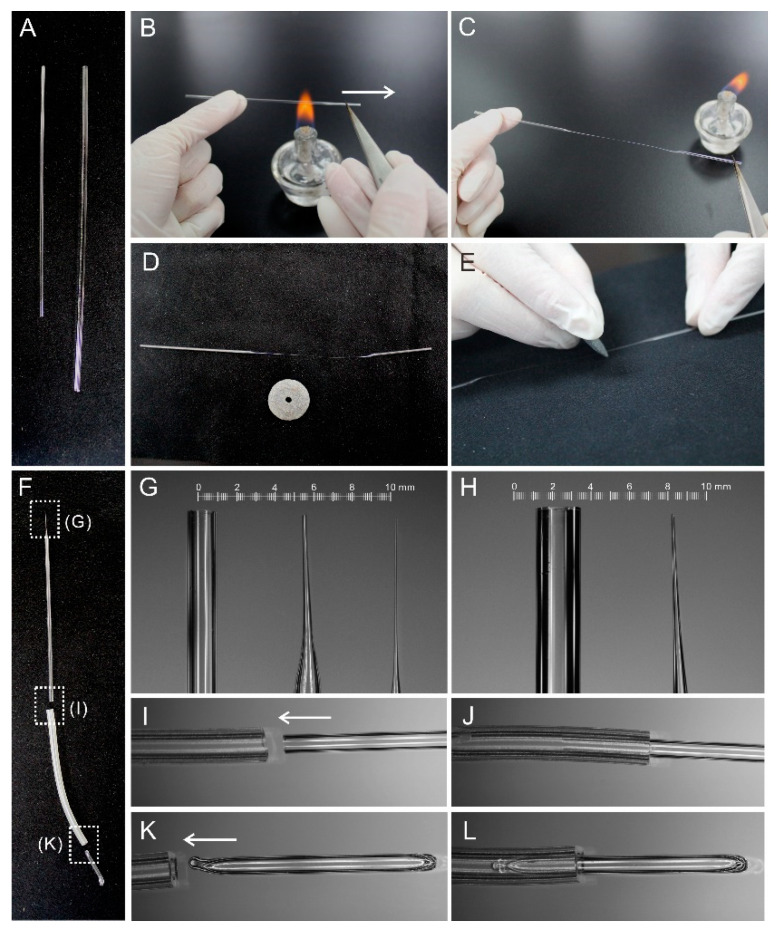
Manual preparation of tools for embryo isolation and TUNEL assay. (**A**) Glass microcapillary (left) and glass rod (right); (**B**–**E**) manual preparation of microcapillary or glass needle using a small spirit lamp; (**F**) component of a capillary pipette with latex tubing; (**G**) untreated microcapillary (left), microcapillary tips with a diameter of around 200 μm (middle), and microcapillary tips with a diameter of around 100 μm (right); (**H**) untreated glass rod (left) and fine glass needle with a diameter of around 200 μm (right); (**I**–**L**) assemble of a capillary pipette with latex tubing.

Firstly, hold one end of the glass tube and clamp the other end with tweezers (Figure 2A,B). Secondly, according the different length, place the glass tube on the flame and quickly pull the tweezers horizontally to make the glass tube form a thinner section (Figure 2C), which is the key step and requires trial and error. Then, cut the glass tube carefully with an emery wheel to make the hand-made glass needles (Figure 2D,E). To assemble the hand-made capillary pipette with a latex tubing, we prepare a 4 cm of flexible latex tube and a 1 cm of sealed glass tube (Figure 2F). Check the integrity and diameter of the glass nozzle under a microscope (Figure 2G,H); then choose the microcapillary tips with a diameter of around 200 μm (Figure 2G) for embryo sac collection, and choose another one with a diameter of around 100 μm for embryo collection (Figure 2G). Then, insert the wide end of the glass tube into the latex tube (Figure 2I,J), and insert the sealed glass tube into the other end of the latex tube (Figure 2K,L). Finally, carefully seal the two junctions with Parafilm (Figure 1C).

### 2.2. Collection of Living Embryos

**Figure 3:**
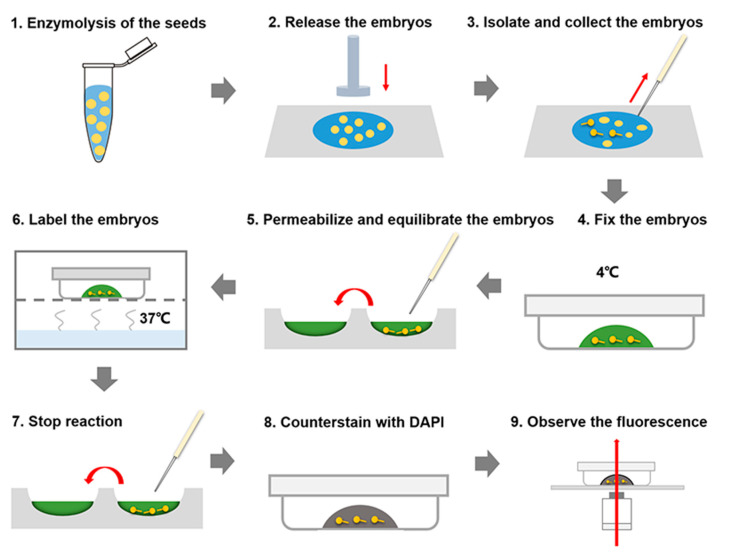
Schematic representation of the assay for the whole mount detection of the suspensor cell death by TUNEL. Steps 1–3 indicate the isolation of living embryos. Steps 4–9 indicate the detection of suspensor programmed cell death (PCD) by TUNEL.

Isolation of living embryos was performed according to the previous protocol [18]. Brief procedures are summarized in the Figure 3. In the step of embryo collection, tobacco embryos after stage 4 could be directly released from the seeds. If it is not very efficient to isolate embryo sacs by grinding the seeds, we can dissect the seeds by fine glass needles (Figure 1B) to release the embryo sacs. Usually, pressing the micropylar end gently by a fine glass needle and cutting on the seed coat by another glass needle are helpful to release the embryo sac from the seed coat. In addition, the treatment of secondary enzymolysis are required to dissect the embryos before stage 4 from the embryo sac, as previously described [18]. Wash the embryos twice with 50 μL of washing buffer, and store them in the washing buffer for subsequent TUNEL analysis. 

### 2.3. TUNEL Assay

Based on the DeadEnd™ Fluorometric TUNEL System, fragmented DNA could be measured by catalytically incorporating fluorescein-12-dUTP at 3′-OH DNA ends via the Terminal Deoxynucleotidyl Transferase, Recombinant, enzyme (rTdT) [13]. The fluorescein-12-dUTP labeled DNA could be visualized directly by fluorescence microscopy.

#### 2.3.1. Fix the Embryos

Firstly, prepare a droplet of 50 μL fresh fixation buffer in the center of a 3.5 cm Petri dish. Then, transfer these isolated embryos carefully into the fixation buffer by a hand-made capillary pipette (Figure 1C), and seal the Petri dish with Parafilm carefully. Fix the embryos for 15 min at 4 °C (Figure 3). 

#### 2.3.2. Permeabilize and Equilibrate the Embryos

During the fixation, add 200 μL of phosphate-buffered saline (PBS) solution into each well of the thick glass slide (Figure 1D). Transfer the embryos carefully into PBS solution by a hand-made capillary pipette to wash the embryos for 5 min at room temperature. Transfer these embryos carefully into another well with the fresh PBS to wash the embryos again. During the second washing, add 200 μL of PBST (PBS containing Triton^®^ X-100) solution into a well of the thick glass slide (Figure 1D). Transfer the embryos carefully into PBST for 5 min at room temperature. After permeabiliztion in the PBST solution, wash the embryos with PBS twice. Add 100 μL of equilibration buffer into a well of the thick glass slide. Transfer the embryos carefully into the equilibration buffer, and incubate them for 5–10 min at room temperature. 

#### 2.3.3. Label the Embryos

While the embryos are incubated in the equilibration buffer, thaw the Nucleotide Mix on ice; keep the Nucleotide Mix and rTdT incubation buffer solution on the ice until use, and protect it from light. The volume of a standard reaction was enough for testing over 150 globular embryos. Then, prepare sufficient TdT reaction mix in the center of a 3.5 cm Petri dish. Transfer the embryos carefully into TdT reaction solution, and carefully seal the Petri dish with Parafilm. Incubate the Petri dish in a humidified chamber for 60 min at 37 °C, and avoid exposure to light from this step forward (Figure 1). 

Prepare a negative control incubation buffer without rTdT by combining 45 μL of equilibration Buffer, 5 μL of Nucleotide Mix and 1 μL of ddH_2_O. This step is optional because the unspecific background could hardly be detected.

If a positive control is desired, treat the embryos with DNase I as the following procedure. Add 100 μL of DNase I buffer to the fixed embryos, and incubate them for 5 min at room temperature. Transfer the embryos into 100 μL of DNase I buffer containing 10 units/mL of DNase I, and incubate them for 10 min at room temperature. After DNase I treatment, wash the embryos with the PBS solution twice. 

During the labeling reaction, add 200 μL of 2 × SSC (Saline-sodium citrate) solution into a well of the thick glass slide. Transfer the embryos carefully in 200 μL of 2 × SSC solution to stop the reaction for 15 min at room temperature. Wash the embryos with the PBS solution twice to remove unincorporated fluorescein-12-dUTP. Stain the embryos in 40 μL of 1 × 4′,6-diamidino-2-phenylindole (DAPI) solution in the dark for 5 min at room temperature. Wash the embryos in the PBS solution twice.

#### 2.3.4. Analyze the Fluorescence

**Figure 4:**
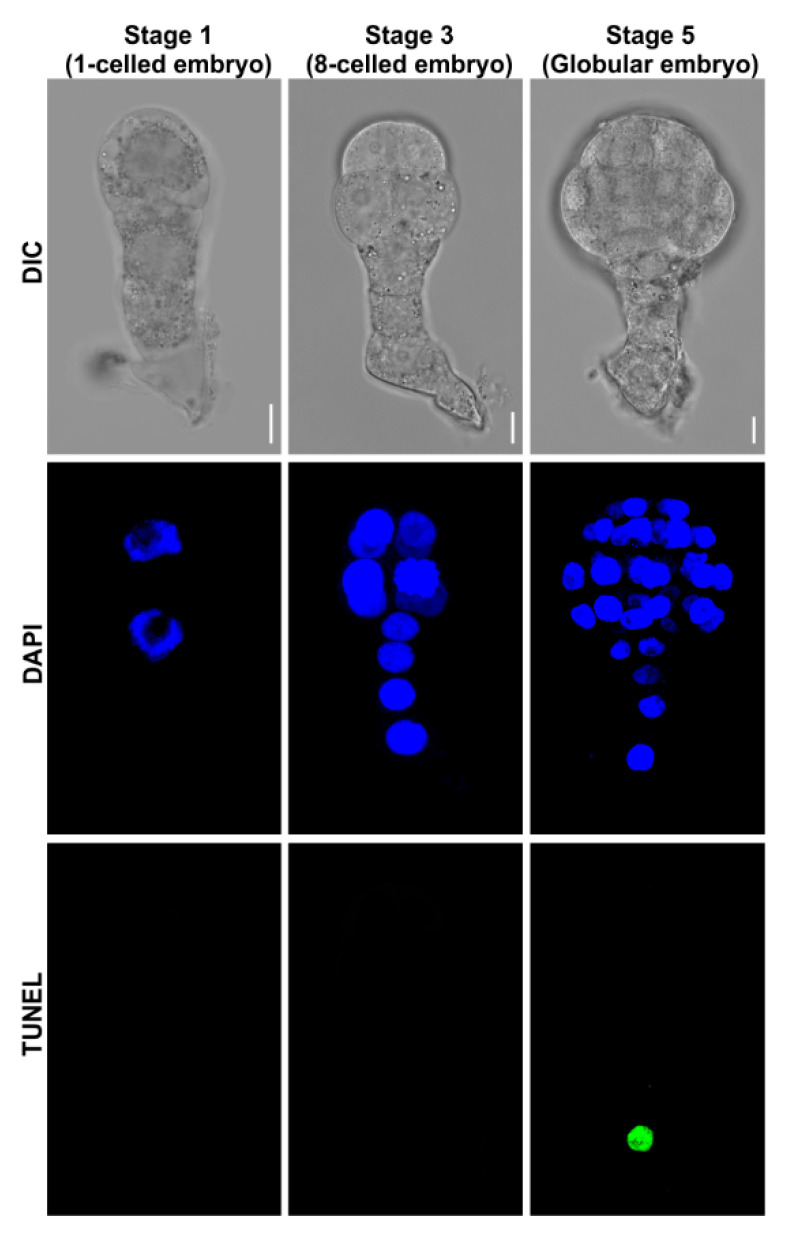
Tobacco embryonic suspensor PCD analyzed using the present protocol. PCD in early embryos at different developmental stages were analyzed. The blue channel indicated embryonic cell nucleus stained with DAPI (4′,6-diamidino-2-phenylindole). The green channel indicated TUNEL-positive suspensor cell. Bar = 10 μm.

The samples were then observed under a confocal microscope, with the following parameter settings: DAPI (λ_ex_ 364 nm; λ_em_ 460 ± 20 nm) and fluorescein (λ_ex_ 488 nm; λ_em_ 520 ± 20 nm) (Figure 4). If the embryos have been labeled with other fluorescence proteins [e.g., GFP (Green fluorescent protein) or YFP (Yellow fluorescent protein)], we suggest to detect the TUNEL signal using In Situ Cell Death Detection Kit, TMR (Tetramethylrhodamine) red (Roche), TUNEL (λ_ex_ 554 nm; λ_em_ 580 ± 20 nm). The protocol is almost the same as mentioned above; see the manufacturer’s manual for more details.

## 3. Materials and Equipment

### 3.1. Plant Materials

*Nicotiana tabacum* var. SR1 plants were grown on natural soil in the greenhouse under a 16 h light/8 h dark photoperiod at 25 °C.

### 3.2. Reagents

NaCl (10019318; Sinopharm Chemical Reagent Co. Ltd., Shanghai, China), KCl (10016318; Sinopharm Chemical Reagent Co. Ltd., Shanghai, China), Na_2_HPO_4_ (10020318; Sinopharm Chemical Reagent Co. Ltd., Shanghai, China), KH_2_PO_4_ (10017618; Sinopharm Chemical Reagent Co. Ltd., Shanghai, China), Sodium citrate (W302600; Sigma, St. Louis, MO, USA), D-Mannitol (M4125; Sigma, USA), Cellulase R-10 (Yakult Pharmaceutical Industry Co. Ltd., Tokyo, Japan), Macerozyme R-10 (Yakult Pharmaceutical Industry Co. Ltd., Japan), Mineral oil (M5904; Sigma, USA), 2-(N-Morpholino) ethanesulfonic acid hydrate (MES) (M8250; Sigma, USA), Paraformaldehyde (158127; Sigma, USA), Triton^®^ X-100 (0694; Amresco, Solon, OH, USA), DAPI (D9542; Sigma, USA), DeadEnd^TM^ Fluorometric TUNEL System (G3250; Promega, Madison, MI, USA), RQ1 RNase-Free DNase (M6101; Promega, USA), and In Situ Cell Death Detection Kit, TMR red (12156792910; Roche, Basel, Switzerland) (if necessary). 

### 3.3. Equipment

Inverted microscope (CK2; OLYMPUS, Tokyo, Japan), Confocal microscope (SP8; Leica, Wetzlar, Germany), fine tweezer, glass microscope slide (80312; CITOTEST, Haimen, China), Glass microcapillary (2177401; HIRSCHMANN, Eberstadt, Germany), fine glass rod (2.5 mm × 130 mm, custom-made; YUNCHENG, China), flexible latex tube (inner diameter = 1.5 ± 1 mm, outer diameter = 2.3 ± 1 mm; DAOGUAN, Shanghai, China), emery wheel (3.2 mm × 21 mm; JIAKANG, China), parafilm M (PM-996; Bemis, Neenah, WI, USA), hand-made glass pestle (Figure 1A), hand-made glass needles (Figure 1B), hand-made capillary pipette with latex tubing (Figure 1C), Petri dishes for microscope observations (3.5 cm), thick glass slide with double well concavity (5-mm, custom-made; HUICHENG, Taizhou, China) (Figure 1D), humidified chamber (a light-proof box with moisture gauze to keep wet), and incubator (LRH-400A; RUIHUA, Wuhan, China). 

### 3.4. Solutions

Washing buffer: 13% D-mannitol, 0.058% MES, pH 5.8. Enzyme buffer I: 1% Cellulase R-10 and 0.8% Macerozyme R-10 dissolved in the washing buffer, pH 5.8. Filter-sterilize the enzyme buffer with a 0.22-μm filter, and make single-use aliquots. Store at −20 °C. Enzyme buffer II: 0.25% Cellulase R-10 and 0.2% Macerozyme R-10 dissolved in the washing buffer, pH 5.8. Filter-sterilize the enzyme buffer with a 0.22-μm filter, and make single-use aliquots. Store at −20 °C. Phosphate-buffered saline (PBS): 137 mM NaCl, 2.7 mM KCl, 10 mM Na_2_HPO_4_, and 1.76 mM KH_2_PO_4_ in ddH_2_O, pH 7.4. Fixation buffer: 4% Paraformaldehyde in PBS. Make fresh 4% paraformaldehyde in the PBS solution for each experiment. It is necessary to warm solution to dissolve paraformaldehyde at 60 °C. Preparations should be carried out in a fume hood. Store the fixation buffer at 4 °C for up to 1 week. PBST: 0.2% Triton^®^ X-100 in PBS. Equilibration buffer (a component in the DeadEnd^TM^ Fluorometric TUNEL System). TdT reaction solution: Add 45 μL of equilibration buffer, 5 μL of Nucleotide Mix which contains fluorescein-12-dUTP, and 1 μL of rTdT per one reaction. SSC (20×): 3 M NaCl, 0.3 M sodium citrate in ddH_2_O, pH 4.5. SSC (2×): Warm 20 × SSC to room temperature to ensure that all salts are in solution. Dilute 1:10 with ddH_2_O before use to generate 2 × SSC. DAPI (1000×): 1 mg DAPI in 1 mL ddH_2_O. Store in the dark at 4 °C for 2 to 3 weeks. DAPI solution (1×): Dilute DAPI (1000×) with PBS before use to generate 1 × DAPI solution.

## 4. Discussion

Suspensor is a terminally differentiated embryonic organ, which helps the embryo to fix in the seed and transfers nutrients and plant hormones to the embryo for normal development [1,2]. Previous research demonstrated that suspensor degeneration is a kind of typical PCD. Therefore, we could study the molecular mechanism regulating plant PCD using the suspensor as a model system [2]. TUNEL assay is one of important methods to study PCD in both animals and plants. Although TUNEL assay has been applied to investigate stress induced-PCD and developmental PCD in plants for years [21,22,23], it is still difficult to analyze suspensor PCD using the traditional TUNEL assay methods. Based on our previous report about the isolation of living early embryos [18], here, we described a detailed method for analyzing suspensor PCD via TUNEL using limited early embryos. The equipment described here consists of an inverted microscope, glass microcapillary, fine glass rod, and flexible latex tube (Figure 1). In addition, the key hand-made tools are easy to assemble according to the introduction (Figure 2). This basic setup has been proven to be efficient and reliable in different plants [18,20]. Compared with the other available methods, this procedure offers several advantages: (i) the spatial-temporal characters of suspensor PCD could be quickly detected within 5–6 h, (ii) it can be easily adopted by other researchers due to the simple setup, (iii) it requires affordable equipment for the basic setup, and (iv) this method could also be useful for rapid detection of cell death of abortive embryos. Nevertheless, one of the main difficulties is that it requires practicing it over and over again to ensure the quick isolation and collection of living early embryos under an inverted microscope.

## 5. Conclusions

In conclusion, we developed a detailed protocol for detecting suspensor PCD via TUNEL. Combined with the isolation of living embryo, this method will be widely applied to investigate the spatial-temporal characters of suspensor PCD in different plants.

## References

[1] Kawashima T., Goldberg R.B. (2010). The suspensor: Not just suspending the embryo. Trends Plant Sci..

[2] Peng X., Sun M.-X. (2018). The suspensor as a model system to study the mechanism of cell fate specification during early embryogenesis. Plant Reprod..

[3] Liu Y., Li X., Zhao J., Tang X., Tian S., Chen J., Shi C., Wang W., Zhang L., Feng X. (2015). Direct evidence that suspensor cells have embryogenic potential that is suppressed by the embryo proper during normal embryogenesis. Proc. Natl. Acad. Sci. USA.

[4] Zhao P., Zhou X.-M., Zhang L.-Y., Wang W., Ma L.-G., Yang L.-B., Peng X.-B., Bozhkov P.V., Sun M.-X. (2013). A bipartite molecular module controls cell death activation in the basal cell lineage of plant embryos. PLoS Biol..

[5] Bozhkov P.V., Filonova L.H., Suarez M.F. (2005). Programmed cell death in plant embryogenesis. Curr. Top. Dev. Biol..

[6] Bozhkov P.V., Suarez M.F., Filonova L.H., Daniel G., Zamyatnin A.A., Rodriguez-Nieto S., Zhivotovsky B., Smertenko A. (2005). Cysteine protease mcII-Pa executes programmed cell death during plant embryogenesis. Proc. Natl. Acad. Sci. USA.

[7] Smertenko A.P., Bozhkov P.V., Filonova L.H., von Arnold S., Hussey P.J. (2003). Re-organisation of the cytoskeleton during developmental programmed cell death in *Picea abies* embryos. Plant J..

[8] Filonova L.H., Bozhkov P.V., Brukhin V.B., Daniel G., Arnold S.V. (2000). Two waves of programmed cell death occur during formation and development of somatic embryos in the gymnosperm, Norway spruce. J. Cell Sci..

[9] Bozhkov P.V., Filonova L.H., Suarez M.F., Helmersson A., Smertenko A.P., Zhivotovsky B., von Arnold S. (2004). VEIDase is a principal caspase-like activity involved in plant programmed cell death and essential for embryonic pattern formation. Cell Death Differ..

[10] Wredle U., Walles B., Hakman I. (2001). DNA fragmentation and nuclear degradation during programmed cell death in the suspensor and endosperm of *Vicia faba*. Int. J. Plant Sci..

[11] Lombardi L., Ceccarelli N., Picciarelli P., Lorenzi R. (2007). DNA degradation during programmed cell death in *Phaseolus coccineus* suspensor. Plant Physiol. Biochem..

[12] Lombardi L., Ceccarelli N., Picciarelli P., Lorenzi R. (2007). Caspase-like proteases involvement in programmed cell death of *Phaseolus coccineus* suspensor. Plant Sci..

[13] Gavrieli Y., Sherman Y., Ben-Sasson S.A. (1992). Identification of programmed cell death in situ via specific labeling of nuclear DNA fragmentation. J. Cell Biol..

[14] Rath N.C., Huff W.E., Bayyari G.R., Balog J.M. (1998). Cell death in avian tibial dyschondroplasia. Avian Dis..

[15] Wang W., Xiong H., Lin R., Zhao N., Zhao P., Sun M.-X. (2018). VPE-like protease NtTPE8 exclusively expresses in the integumentary tapetum and is involved in seed development. J. Integr. Plant Biol..

[16] Shi C., Luo P., Du Y.-T., Chen H., Huang X., Cheng T.-H., Luo A., Li H.-J., Yang W.-C., Zhao P. (2019). Maternal control of suspensor programmed cell death via gibberellin signaling. Nat. Commun..

[17] Luo A., Zhao P., Zhang L.Y., Sun M.-X. (2016). Initiation of programmed cell death in the suspensor is predominantly regulated maternally in a tobacco hybrid. Sci. Rep..

[18] Zhao P., Zhou X.-M., Shi C., Sun M.-X. (2020). Manual isolation of living early embryos from tobacco seeds. Methods Mol. Biol..

[19] Palovaara J., Saiga S., Weijers D. (2013). Transcriptomics approaches in the early Arabidopsis embryo. Trends Plant Sci..

[20] Zhou X.-M., Shi C., Zhao P., Sun M.-X. (2019). Isolation of living apical and basal cell lineages of early proembryos for transcriptome analysis. Plant Reprod..

[21] Tripathi A.K., Pareek A., Singla-Pareek S.L. (2016). A NAP-Family Histone chaperone functions in abiotic stress response and adaptation. Plant Physiol..

[22] Biswas M.S., Mano J.I. (2015). Lipid peroxide-derived short-chain carbonyls mediate hydrogen peroxide-induced and salt-induced programmed cell death in plants. Plant Physiol..

[23] Fendrych M., Van Hautegem T., Van Durme M., Olvera-Carrillo Y., Huysmans M., Karimi M., Lippens S., Guérin C.J., Krebs M., Schumacher K. (2014). Programmed cell death controlled by *ANAC033*/*SOMBRERO* determines root cap organ size in Arabidopsis. Curr. Biol..

